# CTPA for clinically suspected pulmonary emboli in oncology patients

**DOI:** 10.1186/1470-7330-14-S1-P27

**Published:** 2014-10-09

**Authors:** RC Brook, B Shepherd, J Smart, E Rutherford, K Tung

**Affiliations:** 1 Radiology Department, University Hospital Southampton, Southampton, UK

## Aim

Pulmonary embolism (PE) is a known cause of morbidity and mortality in oncology patients. Whilst much of the literature focuses on the detection of incidental PE in this cohort of patients (approximately 4%), little is written about the rate of positive CT Pulmonary Angiograms (CTPA) when PE is clinically suspected. We wanted to assess the rate of positive CTPA specifically amongst oncology patients with suspected PE in our teaching hospital.

## Methods

We retrospectively analysed all consecutive CTPAs carried out for clinically suspected PE over a 12 month period in all patients under our oncology services.

## Results

A total of 230 oncology patients had a CTPA to exclude PE and 40 were positive (17.4%). Of the positive CTPAs, most (31 scans, 77.5%) showed multiple PEs. The commonest cancer patients to undergo CTPA were lung (38), breast (34) and lymphoma (26) patients, and the highest incidence of positive CTPA were in breast (7/34), ovarian (2/9) and pancreatic (2/5) carcinoma. CTPAs carried out in colorectal carcinoma patients had the lowest probability of being positive (1/15).

## Conclusion

The published rate of positive CTPA for clinically suspected PE in the general population is 15-20%. Our study shows that despite the widely held view that oncology patients are more prone to pulmonary thromboembolic disease, the incidence of positive CTPA is in fact similar to the general population. Our threshold for imaging oncology patients should therefore be the same as in the general population.

